# Prevalence and risk factors of geohelminths in primary schools children aged 5 to 15 years in the city of Moundou, southwestern Chad

**DOI:** 10.1016/j.parepi.2023.e00330

**Published:** 2023-11-28

**Authors:** Noumedem Anangmo Christelle Nadia, Yamssi Cedric, Adam Makine Ibrahim, Simeni Njonnou Sylvain Raoul, Gamago Nkadeu Guy-Armand, Tako Djimefo Alex Kevin, Kamga Fouamno Henri Lucien

**Affiliations:** a Department of Microbiology, Haematology and Immunology, Faculty of Medicine and Pharmaceutical Sciences, University of Dschang, P.O. Box 96, Dschang, Cameroon; b Department of Biomedical Sciences, Faculty of Health Sciences, University of Bamenda, P.O. Box 39, Bambili, Cameroon; c Department of Internal Medicine and Specialties, Faculty of Medicine and Pharmaceutical Sciences, University of Dschang, P.O. Box 96, Dschang, Cameroon; d Department of Animal Biology, Faculty of Science, University of Dschang, P.O. Box 067, Dschang, Cameroon; e Department of Animal Organisms, Faculty of Science, The University of Douala, P.O. Box 24157, Douala, Cameroon

**Keywords:** Prevalence, Geohelminthiasis, Risk factors, Moundou, Chad

## Abstract

Geohelminthiases are endemic in Chad and constitute a serious public health problem. This study aimed at determing the prevalence and risk factors of intestinal geohelminthiasis in children aged 5–15 years in the city of Moundou, Chad. This was a cross-sectional and descriptive study carried out in the city of Moundou. A total of 333 pupils participated in this study and it included children aged from 5 to 15 years attending three public primary schools in Moundou. A questionnaire was administered to each student after obtaining Informed Consent from either parent. Stool samples were collected in a sterile container and, the formalin-ethyl ether concentration technique was used to identify parasite. Parasitic load was assessed using the Mc Master cell method. The collected data were analyzed using Excel; Word 2016 and SPSS 20 software. An overall prevalence of 16.52% was obtained, 9.3% for *Trichuris trichiura,* 6.9% for *Ascaris lumbricoides*, and 1.2% for Hookworms. Male participants were more infected (67.24%) than females (32.76%). The age group]9–13] was the most infected (53.44%), followed by the age group [5–9](44.83%) and finally the age group]13–15] (1.73%). The Ouhoud school was the most infected (55.17%) followed by the Adoum Dallah school (39.66%) and finally the Centre school (5.17%). However, no statistically significant difference between gender and geohelminthiasis infection was recorded (*p* > 0.05). Regarding risk factors, statistical analysis showed that age group]9–13] (OR = 1.997 at 95% CI at [1.085–3.677]), Central Public School (OR = 1.55 at 95% CI at [0.63–2.46]), tap water (OR = 29 at 95% CI at [20.89–38.70]), not maintaining latrines (OR = 2.37 at 95% CI at [0.62–3.78]), and maintenance of latrines by pupils (OR = 1.5 at 95% CI at [0.63–2.46]) were risk factors. This study shows a high prevalence of geohelmenthiasis among children of three primary schools in Moundou, Chad. Although males were more infected than female there was no significant difference between gender and geohelminth infections (*p* = 0.114). was no gender difference. Identified risk factors of geohelmenthiasis infections among the study population were: age between 9 and 13 years, school water consumption, the use of unmaintained latrines and latrines maintained by students. Surveillance of geohelminthiases and hygiene should be intensified to reduce the pathological risk related to these parasites in Chad.

## 1 Introduction

Geohelminths are intestinal nematodes that infect humans and are transmitted mainly through contaminated soils where hygienic conditions are poor (Nkouayep et al., 2017). Infection by these intestinal parasites is related to poverty and the highest prevalence has been observed in developing countries where hygiene and sanitation conditions are inadequate (Diongue et al., 2017). The World Health Organization (WHO) estimates that >1.5 billion people are infected with geohelminths worldwide (Benzalim, 2010). *Ascaris lumbricoides, Ancylostoma duodenale, Necator americanus,* and *Trichuris trichiura* are the most common species infecting humans (Ngui et al., 2011). Children aged 5–15 years are the most vulnerable group of people infected with the disease(Teshale et al., 2018). >568 million children aged 5–15 years live in areas where there is a widespread transmission of these parasites (Mehraj et al., 2008). In 2016, it was estimated that 40% of the population in Africa would be exposed to geohelminths and they remain a major public health issue in endemic countries such as Chad (Mascarini-Serra, 2011). The World Health Organization designates tropical and subtropical regions as a focus for geohelminthiasis, particularly in poor communities that do not have access to safe drinking water for satisfactory use (WHO, 2002). Thus,Tchad, being a subtropical country, and due to its bioclimatic biodiversity, favors the development of numerous human parasites, including geohelminths.Geohelminthiasis is a water-dependent disease that is a serious health problem in hot regions (Dankoni and Tchuenté, 2014). In this context, the risk of contamination is a real problem in the city of Moundou, located in southern Tchad. In this city, a large part of the population has no access to portable drinking water and depends on the city's rivers and wells. Despite all this information, the diagnosis of these parasitic worms is not done in a regular and systematic way, especially as to the best of our knowledge this is the first research done in the city of Moundou. We believe it would be useful to know the health status of the population with respect to geohelminthiasis in children aged between 5 and 15 years in the town of Moundou in TChad.

## 2 Material and methods

### 2.1 Study site

The study was conducted in Chad, a Central African country with a surface area of 1,284,000 km^2^, more precisely in the city of Moundou, capital of the Logone Occidental Region and the Lac Wey Department, located in the extreme south-west of the country, 478 km south of the capital N'Djamena. Samples were collected in three public Primary Schools: the Centre Primary School, Ouhoud Primary School, and the Adoum Dallah Primary School in Moundou.

### 2.2 Study population and sample size

The city of Moundou is an economic metropolis and was chosen as a study site because the large-scale trade there had given room to environmental pollution of the streets and markets by household waste. Our study population was randomly recruited from three schools in the city. These Primary Schools were: Centre Primary School, Ouhoud Primary School, and Adoum Dallah Primary School.The sample size was calculated using the Lorenz formula (StatCalc from EPI Info® software). Using the prevalence of 50.1% in N'Djamena, with 80% power to detect significant associations or differences and an accepted margin of error of 5%, the minimum sample size estimate was 384 participants.

### 2.3 Inclusion and exclusion criteria

All Pupils aged 5–15 years, attending one of these three schools, and whose parents signed the Informed Consent form were included. Those excluded were all pupils who just received anthelminthics within the last three months.

### 2.4 Data and faecal sample collection

In each primary school, children were randomly selected. After selecting children who met the inclusion criteria, the consent form was returned to each parent via the participant. Children aged 10 years and above were asked to complete the questionnaire directly, while children under 10 years were assisted by the teacher in answering the questions. The questionnaire submitted by the participants consisted of two parts. The first part was about socio-demographic data (age, gender, level of education, neighborhood, and religion), and the second part was about risk factors (knowledge, hand and food hygiene, and type of water consumed). After completing the questionnaires, they were given sterile containers and instructions on how to collect the samples (first stool in the morning, collect a considerable amount to avoid contact with the ground). The samples were collected in the morning at 7:30 a.m. These samples were labeled, formalized at 10%, and sealed to preserve the parasite forms. After collection, the samples were transported to the Laboratory of the Faculty of Medicine and Pharmaceutical Sciences, University of Dschang, for parasitological analysis.

### 2.5 Qualitative parasitological examination

Parasitological diagnosis was carried out as described by the World Health Organization (Mondiale De La Santé, 2021). Briefly, about 2 g of faeces were emulsified in a 250 mL beaker and 3 to 4 mL of 10% formalized water were added. The mixture was emulsified for about 1 min. The sieved suspensions were centrifuged for 2–3 min. About 4 mL of diethyl ether was added to the conical tube and shakened well for 1 min. A cotton handkerchief was wrapped around the top of the conical tube and the cap was loosened very gently, then the cap was tightened. The conical tube containing the suspension was immediately centrifuged and helminth eggs or larvae settled to the bottom of the tube and the faecal debris accumulated in a layer between the diethyl ether and 10% formalin.

### 2.6 Quantitative examination

The McMaster technique, as described by the World Health Organization (Mondiale De La Santé, 2021), was used to express parasite load. Briefly, 2 g of stool were diluted in 28 mL of flotation solution and centrifuged at 1500 rpm for 3 min. After pouring out the supernatant, Malassez cells were filled with a pipette. The parasite load was equal to the number of eggs counted in the two Malassez cells, multiplied by 50.

### 2.7 Data analysis

Statistical analysis of the collected data was performed using Word 2016, Excel 2016, and SPSS 20 software. Data were expressed as headcount and percentage. Multivariate logistic regression analysis was performed to identify the main risk factors, Chi^2^-square test of independence was used to test the association between the different risk factors and soil-transmitted helminths. A *P*-value of <5% (*P* < 0.05) with an Odds Ratio > 1 was considered statistically significant at 95% confidence intervals.

## 3 Results

**Fig. 1:**
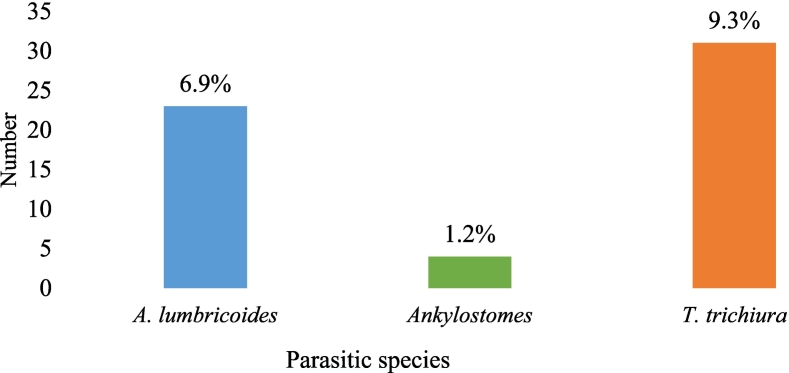
Prevalence of geohelminth species.

**Fig. 2:**
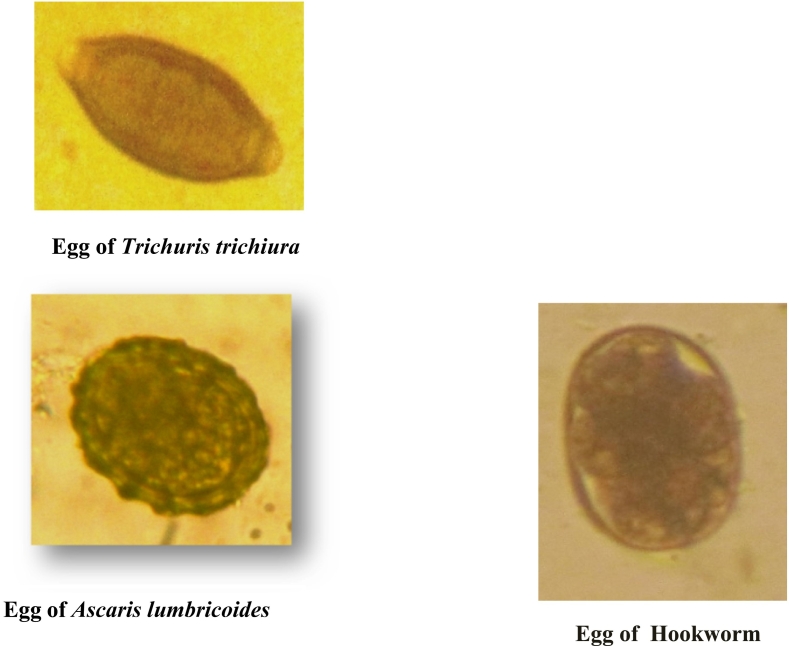
Pictures of identified eggs.

The overall prevalence of geohelminthiasis in this study was 16.52% (55 students who harbored at least one parasite species). Fig. 1 shows the prevalence of geohelminthiasis according to species. It follows from the analysis of this figure that 9.30% of the students were infected with *Trichuris trichiura,* 6.90% with *Ascaris lumbricoides,* and 1.20% with Hookworms. Fig. 2 shows the pictures of identified eggs in this study.

Table 1 shows the prevalence according to gender, age, and primary school. It shows that there was no significant difference between gender and geohelminth infections (*p* = 0.114). While significant differences were observed by age group (p˂0.05) and primary school (p˂0.05).

**Table 1:** Prevalence of geohelminthiasis according to gender, age group, and school.

Modality	Positive numbers *n* = 58(%)			Total	P-Value

	*A. lumbricoides*	*Ankylostomes*	*T. trichiura*		
Gender					
Female	9 (15.51)	1 (1.73)	9 (15.51)	19 (32.75)	P = 0.114
Male	14 (24.14)	3 (5.17)	22 (37.94)	39 (67.25)	
Age range					
[5–9]	13 (22.41)	1 (1.73)	12 (20.68)	26 (44.83)	*P* = 0.003
[9–13]	10 (17.24)	2 (3.44)	19 (32.75)	31 (53.44)	
[13–15]	0	1 (1.73)	0	1 (1.73)	
School					
Adoum Dallah	9 (15.51)	0 (0)	14 (24.13)	23 (39.66)	*P* = 0.01
Centre	1 (1.21)	0	2 (2.43)	3 (5.17)	
Ouhoud	13 (22.41)	4 (6.89)	15 (25.86)	32 (55.17)	
Total	23 (39.65)	4 (6.90)	31 (53.45)	58 (100)	

Table 2 presents the prevalence according to origin of water consumed and hygiene. There is no statistically significant difference between hand and food hygiene (*p* > 0.05). While there was a statistically significant difference in water consumption at school (*p* < 0.05).

**Table 2:** Prevalence of geohelminthiasis according to hygiene and eating habits.

Variables	Positive n = 58	*A.lumbricoides* n (%)	Ankylostomes n (%)	*T. trichiura* n (%)	P -value
Water consumed at school					p > 0.05
Drilling	26	10 (38.46)	0 (0)	16 (61.54)	
Well	32	13 (40.62)	4 (12.5)	15 (46.88)	
Hand washing					p > 0.05
Yes	25	19 (76)	1 (4)	5 (20)	
No	33	4 (12.12)	3 (9.09)	26 (78.78)	
Food washing					p < 0.05
Yes	58	23 (39.65)	4 (6.90)	31 (53.44)	
No	0	0 (0)	0 (0)	0 (0)	

Table 3 shows the parasite load according to sex, level of education, and age group. It can be seen that although parasite load varied according to sex, age, and education, no differences were noted. All the parasite loads were low, with <3000 eggs per gram of stool.

**Table 3:** Parasitic load according to gender, age group, and level of education.

Variable	Modality	Species	Mean infestation intensity		
			Low	Moderate	Heavy
Gender	Male	*Ascaris lumbricoides*	2715 ± 2736,898	–	–
		*Trichuris trichiura*	1286.36 ± 1146.338	–	–
		*Ankylostomes*	1400 ± 346.410	–	–
	Female	*Ascaris lumbricoides*	1834.44 ± 1486.835	–	–
		*Trichuris trichiura*	850 ± 1089.725	–	–
		Ankylostomes	600	–	–
Level of education	Class 4–6	*Ascaris lumbricoides*	2531.00 ± 2463.395	–	–
		*Trichuris trichiura*	1400 ± 346.410	–	–
		Ankylostomes	1200 ± 489.898	–	–
	Class2–3	*Ascaris lumbricoides*	1300 ± 173.205	–	–
		*Trichuris trichiura*	1500 ± 424.264	–	–
		Ankylostomes	0	–	–
Age range	[5–9]	*Ascaris lumbricoides*	2718.00 ± 2675.041	–	–
		*Trichuris trichiura*	1379.55 ± 1153.015	–	–
		Ankylostomes	900 ± 424.264	–	–
	[9–13]	*Ascaris lumbricoides*	1718.75 ± 1410.151	–	–
		*Trichuris trichiura*	1226.67 ± 1334.738	–	–
		Ankylostomes	1200	–	–
	[13–15]	*Ascaris lumbricoides*	0	–	–
		*Trichuris trichiura*	0	–	–
		Ankylostomes	0	–	–

Table 4 presents the risk factors for infection. It shows that age group]9–13] (OR = 1.997 at 95% CI at [1.085–3.677]), Central Public School (OR = 1.55 at 95% CI at [0.63–2.46]), tap water (OR = 29 at 95% CI at [20.89–38.70]), not maintaining latrines (OR = 2.37 at 95% CI at [0.62–3.78]), and maintenance of latrines by pupils (OR = 1.5 at 95% CI at [0.63–2.46]) were risk factors.

**Table 4:** Distribution of geohelminthiasis according to risk factors.

Risk factors	Geohelminthiasis Positive (%)	Odds ratio	CI 95%	P-value
Gender				
Female	18 (32.73)	0.61	0.276–1.129	0.114
Male	37 (67.27)	0.48	0.311–1.577	0.116
Age range				
[5–9]	17 (30.91)	0.616	0331–1.144	0.122
[9–13]	37 (67.27)	1.997	1.085–3.677	0.020*
[13–15]	1 (1.82)	0.239	0.031–1.818	0.134
Level of education				
Class 2–3	5 (9.09)	0.371	0.142–0.973	0.571
Class 5–6	50 (90.90)	0.371	0.142–0.973	0.587
Schools				
Adoum Dallah	23 (41.82)	15.23	10.30–21.93	0.42
Centre	3 (5.46)	1.55	0,63-2,46	0.013*
Ouhoud	29 (52.72)	0.82	0,31-2,59	0.009
Latrine maintenance service				
Maintenance agent	3 (5.45)	3.65	1.17–10. 83	2.353
By the students	23 (41.82)	1.55	0.63–2.46	0.031*
No maintenance	29 (52.73)	2.37	0.62–3.78	0.001*
Hand washing before eating				
No	10 (18,18)	12.19	6.64–21.31	0.297
Yes	45 (81,82)	17.92	13.63–23.20	0.147
Water consumed at school				
Drilling	26 (47.27)	11.15	7.69–15.91	0,231
Well	29 (52.73)	29.00	20.89–38.70	0.001*
Washing food (fruits, vegetables) before eating				
No	0 (0)	100	0.00–0.00	–
Yes	55 (100)	16,71	13.04–21.17	0.827

No association of risk of geohelminthiasis infection was found between gender, level of educational, hand washing, and fruit washing before eating. However, water consumed at school increased the risk of geohelminthiasis by 7.53 times. Furthermore, latrine maintenance service was significantly (*p* < 0.001) correlated with geohelminthiasis infection. However, the Ouhoud Primary School showed the highest prevalence, followed by Adoum Dallah Primary School and finally the Centre Primary School, 55.17%, 39.66%, and 5.17% respectively. This study showed a significant difference between well water consumed at school, study sites (Centre, school, and Ouhoud), latrines not maintained, latrines maintained by pupils, and geohelminthiasis infection (*p* < 0.05).

## 4 Discussion

The results of this cross-sectional study showed a high prevalence of geohelminthiasis infection of 16.52%. Although several studies have been carried out on this topic in Chad, this is the first on analysis of geohelminth infection in the City of Moundou.

Children aged 5 to 15 years were infected with geohelminths in the city of Moundou. This prevalence was lower than that of a previous study conducted by (Hamit et al., 2008) in N'Djamena in 2008, which showed a prevalence of 51% of helminth infections in subjects aged 0 to 70 years. This prevalence seems to decrease over time: 51% in 2008 and 90% in 1990. This decrease could be due to an evaluation of the standard of living, improved hygienic practices and a policy of systematic deworming of the population. A study conducted by Flaure Edith et al. (2020) showed a prevalence of 41% among children aged 5 to 15 years in the city of Douala, this difference can be explained by the geographical location of Douala near the coast, a higher rainfall than that of Moundou, thus making the population of the city of Douala more vulnerable to parasitic diseases.

However, this result corroborates that of (Ruth et al., 2021) and (Awono-Ambene et al., 2020) in 2021 who obtained a prevalence of 11.6% in Bamendjou (in the Western Region of Cameroon) and 9.6% in Yaounde (the capital city of Cameroon), this prevalence could be justified by the multiple deworming campaigns organized by the Ministry of Public Health.

The overall prevalence of intestinal parasites by gender showed that boys (67.27%) were more infected than girls(37.73%). This difference in prevalence observed amonght gender could be justified by the fact that at an early age, male children are much more interested in playing and are therefore comparatively more exposed to contaminated soil and water, a predisposing factor for infection. These results are in agreement with those of Awono-Ambene et al. (2020) and Tchuem Tchuenté et al. (2003) in Cameroon who showed the predominance of geohelminthiasis in males. Similarly, Flaure Edith et al. (2020) showed a predominance of geohelminthiasis in boys, but with no significant difference between the two sexes. However, these results are contrary to those of Ruth et al. (2021) who observed in Bamendjou, Western region of Cameroon, that more girls were infected.

In general, the highest rates were found in the age groups of 9 to 12 years (67.27%), 5 to 8 years (30.91%), and 13 to 15 years (1.82%). Several studies revealed that children aged 5 to 15 years were the most affected (Hamit et al., 2008),(Novianty et al., 2018; Ruth et al., 2021). This finding could be explained by the fact that this population has a more active community life and observes less hygienic rules, and these results could be further justified by the fact that children in this age group are always in contact with the soil. Children between 13 and 15 years of age were less infected, as children in this age group are relatively more mature and more hygienic.

The most prevalent species of intestinal geohelminths were *Trichuris trichiura* 9.30%, followed by *Ascaris lumbricoides* 6.90% and Ankylostoma 1.20%. These results are similar to those of Almaw (Almaw, 2020) who in 2019 in Indonesia found a prevalence of Hookworms of 3.4% in children aged 5–15 years. The predominance of *Trichuris trichiura* species and *Ascaris lumbricoides* could be explained by the fact that these two geohelminths are much more resistant to climatic factors and have a common mode of contamination in contrast to Hookworms which are much more waterrelated and have a transcutaneous mode of contamination.

We found that the species which was most involved in infection was *Trichuris trichiura;* this is contrary to the results of (Yamssi Cedric et al. (2020), who in a study conducted in the city of Bamenda in the Northwest Region of Cameroon, reported a high (17%) prevalence of Hookworm species and a low (5%) prevalence of *Trichuris trichiura* in children aged 0–14 years. (Awono-Ambene et al., 2020), found a predominance of *Ascaris lumbricoides and Enterobius vermicularis.* This high prevalence in these children with hookworms could be attributed to the fact that they play a lot on the ground barefooted. The soil is known to harbor a good number of eggs of parasites that end up in the body of their host as a result of poor hygienic practices.

This study showed the prevalence of double parasitism. The association of *Trichuris trichiura* + *Ascaris lumbricoides* was 5.76%. This result is similar to that of Ruth et al. (2021), who recorded a prevalence of double parasitism of 8.2%. Similarly, Menan et al. (1997) reported a prevalence of 7.8% of the double parasitism association of *Ascaris lumbricoides + Trichuris trichiura*. Similarly, this study also showed a double parasite association of *Trichuris trichiura + Ascaris lumbricoides.* The coinfection of *Ascaris lumbricoides + Trichuris trichiura* would probably be due to the similarity in their transmission routes (faeco-oral) (Albonico et al., 2003). With respect to the overall predominance of these two species, climate and the soil of the city of Moundou could explain the abundance in this study area.

Indeed, previous studies have also confirmed that open defecation can increase the risk of infection and polyparasitism nearly two-fold (Sumbele et al., 2021). This finding is similar to that of Ramayanti and Ghiffari (2019) who had conducted a study on children aged 5–15 years. Their study showed that latrines and water consumed at school were significant risk factors (Ramayanti and Ghiffari, 2019).

This high prevalence between schools could be justified by the fact that these schools are located in a neighborhood that is frequently flooded during the rainy season and is near the Logone River.

This study showed that the pupils of class 3–4 were more infected and this level corresponds to the age range of 7 to 12 years, this observation is similar to those of Flaure Edith et al. (2020) who showed that the prevalence of intestinal geohelminths was higher in the age group 5 to 11 years in 2020 in the city of Douala. Similarly, (Awono-Ambene et al., 2020) found that children aged from 11 to 13 years are more prone to parasitic infection. This could be explained by the fact that children of this age are actively and constantly playing making them to be always in contact with the contaminated environment or objects.

However, this study has delivered valuable information concerning the prevalence and associated factors of geohelminths in primary school children aged 5 to 15 years in the city of Moundou which will provide baseline information to the Chad Deworming Programme to effectively control these diseases. One of the limitations of this study was the lack of Polymerase Chain Reaction (PCR) molecular technique to confirm the species of parasite identified in this study.

## 5 Conclusion

The overall prevalence of geohelminthiasis infection was 16.52% in the city of Moundou. This study assessed the association of potential risk factors with prevalence of geohelminthiasis. School water consumption, school latrine maintenance service, age group, and attendance site were found to be statistically significant risk factors for geohelminthiasis infection among the children in this study. However, it will be important to extend the study to other schools in the country.

## Funding

The study received no funding from any source or organization.

## Authors' contributions

NACN, YC, SNSR and KFHL conceived the idea and designed the study. AMI, GNGA, and TDAK performed the experiments. YC and GNGA analyzed and interpreted the data. NACN, SNSR, TDAK and YC drafted the manuscript. All authors read and approved the final manuscript.

## Declaration of Competing Interest

The authors declare that they have no competing interests.

## Data Availability

All data generated and analyzed are included in this research article.
